# Exploring Service Providers' Perspectives in Improving Childhood Obesity Prevention among CALD Communities in Victoria, Australia

**DOI:** 10.1371/journal.pone.0162184

**Published:** 2016-10-13

**Authors:** Sheila Cyril, Julie Green, Jan M. Nicholson, Kingsley Agho, Andre M. N. Renzaho

**Affiliations:** 1 School of Social Sciences and Psychology, Western Sydney University, Locked Bag 1797, Penrith, New South Wales, Australia; 2 Department of Epidemiology & Preventive Medicine, School of Public Health and Preventive Medicine, Monash University, Prahran, Victoria, Australia; 3 Raising Children Network, Murdoch Children’s Research Institute and Department of Paediatrics, University of Melbourne, Parenting Research Centre, Level 5/232 Victoria Parade, East Melbourne, Victoria, Australia; 4 Judith Lumley Centre, La Trobe University, Level 3, Melbourne, Victoria, Australia; 5 School of Science and Health, Western Sydney University, Locked Bag 1797, Penrith, New South Wales, Australia; 6 School of Social Sciences and Psychology, Western Sydney University, Locked Bag 1797, Penrith, New South Wales, Australia; Kyoto University, JAPAN

## Abstract

**Background:**

Childhood obesity rates have been increasing disproportionately among disadvantaged communities including culturally and linguistically diverse (CALD) migrant groups in Australia due to their poor participation in the available obesity prevention initiatives. We sought to explore service providers’ perceptions of the key factors influencing the participation of CALD communities in the existing obesity prevention services and the service requirements needed to improve CALD communities’ participation in these services.

**Methods:**

We conducted a qualitative study using focus group discussions involving fifty-nine service providers from a range of services, who are involved in the health and wellbeing of children from CALD groups living in four socioeconomically disadvantaged areas in Victoria, Australia.

**Results:**

Thematic analysis of the data showed three major themes including community-level barriers to CALD engagement in childhood obesity prevention services; service-level barriers to the delivery of these services; and proposed changes to current childhood obesity prevention approaches. Integrating obesity prevention messages within existing programs, better coordination between prevention and treatment services and the establishment of a childhood obesity surveillance system, were some of the important changes suggested by service providers.

**Conclusion:**

This study has found that low CALD health literacy, lack of knowledge of cultural barriers among service providers and co-existing deficiencies in the structure and delivery of obesity prevention services negatively impacted the participation of CALD communities in obesity prevention services. Cultural competency training of service providers would improve their understanding of the cultural influences of childhood obesity and incorporate them into the design and development of obesity prevention initiatives. Service providers need to be educated on the pre-migratory health service experiences and health conditions of CALD communities to ensure equitable delivery of care. Collaborative approaches between health systems, immigrant services, early years’ services and community health services are urgently needed to address obesity-related disparities in Australia.

## Introduction

Obesity is currently the second highest contributor to the burden of disease in Australia, and is one of the nine National Health Priority areas for the Australian government [1]. The 2014–15 National Health Survey data showed that overweight and obesity in children rose from 25.7% in 2011–12 to 27.4% in 2014–15 [2]. Obesity in children is associated with numerous health risks including hypertension, cardiovascular disease, stroke, diabetes, gall bladder disease, cancer, mental illness and predisposition to adulthood obesity [3]. Further, the economic burden of obesity in industrialised countries including Australia is on the rise with the most recent estimate showing that the total cost of obesity in Australia was $8.6 billion as of 2012 and the estimated health and wellbeing cost to individuals from obesity being $47.4 billion in 2011–12, including the impact of obesity on quality of life and length of life [4–6]. Further, obesity prevention alone entails an annual spending of $145 million in Australia [6].

Although, the National Preventative Health Strategy had committed to reducing health inequities by targeting disadvantage, overweight and obesity rates continue to rise among disadvantaged populations including culturally and linguistically diverse (CALD) communities, indigenous populations and low socioeconomic groups, who bear a disproportionate burden of childhood overweight and obesity (32%), compared to children from the mainstream Australian-born English-speaking populations (25%) [7–9]. CALD communities are defined as those people born overseas, in countries other than those classified by the Australian Bureau of Statistics (ABS) as “main English speaking countries” which include New Zealand, the United Kingdom, United States of America, Canada, Ireland and South Africa [10,11]. CALD communities include those who speak a language other than English as their first spoken language and possess limited proficiency in English [10]. As of 2015, nearly 20% of Australia’s population was made up of CALD groups [12]. In the last two decades, numerous obesity prevention initatives implemented in Australia and New Zealand managed to reduce childhood obesity among mainstream populations [13,14] however, they not only failed to achieve obesity reduction when implemented among CALD communities, but also led to an increase in their obesity prevalence rates [15–17]. Indeed, there is growing concern over the existing obesity prevention initiatives ineffectively targeting disadvantaged populations thereby worsening the existing childhood obesity-related disparities in Australia [8,18–21].

There is increasing recognition among policymakers and service providers, that using a cross-cultural multi-pronged approach involving a range of stakeholders from government, health services, schools and early years’ services, is a crucial step in reducing childhood obesity-related disparities [8,22,23]. Past research has shown that engaging key stakeholders who have an ongoing involvement in the health and wellbeing of young children including modifying factors that contribute to child unhealthy weight gain, can achieve obesity reduction [24–28]. Particularly among disadvantaged communities, in addition to determining target populations’ views, [29–31] obtaining key stakeholders’ perspectives on the barriers experienced by these communities in engaging with obesity prevention programs, has been useful in improving their program participation rates [32,33]. In the US, such studies have resulted in improvements in obesity prevention among disadvantaged children [34,35]. However, in Australia, while studies have examined the perspectives of service providers to improve childhood obesity prevention among mainstream Australian populations, no such studies have been conducted to improve childhood obesity prevention among CALD populations [28].

Currently there is a gap in the knowledge of service providers’ perspectives of the various barriers impacting the effective utilisation of childhood obesity prevention services by CALD communities in Australia. Through the lens of service providers, this study aimed to address this evidence gap and enable the identification of key changes to the existing childhood obesity prevention policy and practice, in order to improve the delivery of obesity prevention services to CALD communities in Victoria, Australia. We used a qualitative approach to capture the views of a diverse sample of key service providers on the barriers and facilitators to participation of CALD communities in childhood obesity prevention initiatives. Findings from this qualitative research will enable policymakers and service providers to address the current gaps in childhood obesity prevention service delivery and utilisation among CALD communities.

## Methods

### Study design and setting

We conducted a qualitative study using focus group discussions to explore key stakeholders’ perspectives on the engagement of CALD communities in childhood obesity prevention initiatives, to generate knowledge in an area where there has been minimal prior research and to maximise the effect of group synergies through spontaneous discussions [36]. Focus groups have the advantage of interactions within the groups, which allow greater exploration of individual and shared perspectives on a particular topic thereby opening up a range of views [37,38]. The study participants included service providers involved in the health and wellbeing of children living in the socioeconomically disadvantaged areas of Maribyrnong, Hume, Brimbank and Greater Dandenong in Victoria, with an Index of Relative Socioeconomic Disadvantage score of <1000 per area. More than 50% of the population in these areas are comprised of migrants and refugees [39]. In Hume, 28% spoke a language other than English at home with majority of the migrant and refugee population originating from the Middle East (12%) (Iraq, Lebanon, Afghanistan and Turkey), Albania, Bhutan and Congo [40]. In Brimbank, 43% were from non-English speaking backgrounds with majority from Vietnam (10%), India, Burma, Malta, Philippines, Horn of Africa, Congo and Sri Lanka [40]. In Maribyrnong, 34% were from non-English speaking backgrounds and majority of migrants and refugees originated from Vietnam (9.5%), India, China, Thailand, Burma, Ethiopia and Afghanistan [40]. In Dandenong, 61% spoke a language other than English at home with the majority from South East Asia (16.6%) (Cambodia, East Timor, Indonesia, Laos, Vietnam) India, Srilanka, Sub-Saharan Africa and Afghanistan [41]. The study was approved by the Monash University Human Research Ethics Committee, approval no. CF14/1443–2014000678.

### Participant recruitment

Purposive sampling was used to identify service providers who are actively involved in the health and wellbeing of children and have the potential to influence healthy lifestyle behaviours among children, as identified by the existing literature [24,25,28]. Based on the advice of the study steering committee which included study investigators, city council staff, maternal and child health (MCH) team-leaders, community health centre managers and primary school wellbeing officers, the research staff conducted face to face meetings with city council representatives of child wellbeing in the four study areas to finalise the process of recruitment. School and kindergarten teachers, playgroup facilitators, health promotion officers, MCH nurses, dieticians, school nurses, day-care centre staff and refugee health nurses, who fulfilled the study inclusion criteria, were purposefully recruited by research staff. Interested stakeholders were given a plain language summary of the study and followed up with phone calls to organise a suitable time for the focus group. Participants were included in the study if they were a) currently employed in kindergartens, primary schools, playgroups, childcare centres, kindergartens, city councils, community health centres, MCH services and Refugee Health services; b) currently working in one of the four areas of greater Melbourne, Australia: Maribyrnong, Hume, Brimbank and Greater Dandenong local government areas. Participants were excluded if they were not a stakeholder of the above-mentioned groups and not employed in any one of the four disadvantaged areas.

### Data collection

Data were collected using eight heterogeneous focus groups (two per geographical area) comprising approximately eight participants (group number ranging from 6 to 9). Each focus group was constructed to include a mix of service providers across a range of community and early years’ services in order to obtain diverse views on the topics discussed. Across all the four study areas, care was taken to ensure the similar composition of groups. Written informed consent was obtained from all participants prior to data collection. Focus groups were conducted by the lead author (SC) and co-facilitated by another member of the research team, in centrally situated locations including community centres, city council offices, and public libraries in each study area, which were convenient and accessible to study participants. Each focus group was around 2 hours long and was audio-recorded and transcribed verbatim. Field notes were taken describing the group dynamics and participant interactions. The focus group discussions were guided by a schedule of four topics identified from the existing literature on childhood obesity among CALD communities [42–45]. The focus group schedule was informed by the agency-structure sociological theory which states that human behaviour is governed by the capacity of individuals to make change (agency) and the social, economic and political contexts within which behaviour change takes place (structure) [18,46]. This theory enabled us to develop a schedule which allowed for exploration of both the service providers’ roles as well as the structure and delivery of services pertaining to childhood obesity prevention. Topics were the service providers’ perceptions of: childhood obesity prevention among CALD communities; service provider roles and responsibilities for promoting healthy eating and physical activity behaviours among CALD communities; barriers and facilitators to the effective utilisation of childhood obesity prevention services by CALD communities; and potential solutions at community and system levels to improve the participation of CALD communities in obesity prevention services. The development of the focus group schedule (S1 File) was overseen by the study steering committee. Data were collected until the study steering committee were satisfied that the focus group schedule topics were adequately covered and no new information emerged (data saturation). Transcripts were shown to study participants who verified and approved the content prior to the commencement of analysis.

### Data analysis

We analysed the data for emergent themes based on grounded theory [47] with two researchers AR and SC, following a six-step thematic analysis approach recommended by Braun and Clarke [48] as follows: 1) Familiarisation with the data by reading the field notes and transcripts in detail; 2) Generation of initial codes across the entire set of transcripts and collating data relevant to each code; 3) Grouping codes into potential themes and gathering all relevant data to each theme; 4) Thematic mapping to review the themes and check if they work in relation to the coded extracts; 5) Defining and naming the themes; and 6) Narration of the themes and sub-themes, with selection of quotations from the raw data to exemplify the unique characteristics of participants’ perceptions. Trustworthiness of the data was ensured by adopting the process of member checks wherein the study participants were given the data analysis report to verify the accuracy of the findings based on their actual experiences. Further, iterative questioning using probes was done to elicit detailed data from participants to complement responses and refer to concepts previously raised by participants to establish clarity, and document synergy and contradictions in participants’ responses. Finally, the research team also had frequent discussions with the steering committee members to obtain their ongoing feedback and inputs into the degree of consistency between data coders.

## Results

Fifty-nine service providers participated in the study. Participant characteristics are summarised in Table 1. About one-third of the total sample (36%) were service providers from early years’ services including schools, kindergartens and playgroups; 32% were service providers from MCH services and city councils; and 32% were from community health centres (nurses, dieticians and health promotion officers).

**Table 1:** Demographic characteristics of the study population.

	Greater DandenongN (%) (n = 15)	Brimbank N (%) (n = 14)	Hume N (%) (n = 15)	MaribyrnongN (%) (n = 15)	Total N (%) (n = 59)
**Age** mean (SD)	39 (2.9)	43 (1.8)	41 (2.2)	48 (1.5)	43 (2.5)
**Gender**					
Male	3 (20%)	2 (14%)	3 (20%)	2 (13%)	10 (17%)
Female	12 (80%)	12 (86%)	12 (80%)	13 (87%)	49 (83%)
**Employment**					
School teacher	2(13%)	2(15%)	1(8%)	2(13%)	7(12%)
School nurse	1 (7%)	1 (14%)	1 (8%)	1 (7%)	4 (7%)
Community health nurse	2 (13%)	2 (14%)	1 (7%)	1 (7%)	6 (10%)
Dietician	2 (13%)	1 (8%)	2 (14%)	2 (13%)	7(12%)
Health Promotion Officer	2 (13%)	2 (14%)	1 (8%)	1 (7%)	6 (10%)
City council staff	2 (13%)	2 (14%)	2 (13%)	2 (13%)	8 (13%)
MCH nurse	1 (7%)	2 (14%)	2 (13%)	2 (13%)	7(12%)
Refugee health nurse	1 (7%)	0 (0%)	1 (8%)	2 (13%)	4 (7%)
Kindergarten teacher	0 (0%)	1 (7%)	2 (7%)	1 (7%)	4 (7%)
Playgroup facilitator	2 (14%)	1 (7%)	2 (13%)	1 (7%)	6 (10%)
**Education**					
High School	2 (13%)	2 (14%)	0 (0%)	2 (13%)	6 (10%)
Diploma/trade	5 (33%)	4 (29%)	5 (34%)	5 (33%)	19 (32%)
Bachelor degree	4 (27%)	6 (43%)	6 (40%)	5 (33%)	21 (36%)
Master’s degree	4 (27%)	2 (14%)	2 (13%)	2 (13%)	10 (17%)
PhD	0 (0%)	0 (0%)	2 (13%)	1 (8%)	3 (5%)

Three major themes emerged from the focus group data and included a) community-level barriers to the engagement of CALD communities in childhood obesity prevention services; b) service-level barriers to the delivery of childhood obesity prevention services; and c) proposed changes to current childhood obesity prevention approaches. Each major theme included a number of minor themes, where the perspectives of service providers unique to each service, as well as the perspectives of the entire group including consensus and disagreements on various topics have been narrated.

### Community-level barriers to the engagement of CALD communities in childhood obesity prevention services

#### Cultural beliefs on childhood obesity among CALD groups

Service providers discussed that cultural beliefs on childhood obesity among CALD groups, including their lack of understanding of childhood obesity, its risk factors and its consequences, and their cultural misconceptions around obesity prevention, affected their utilisation of obesity preventive services.

*”…many of the CALD parents talk about their beliefs such as “obesity is in our genes and cannot be altered” which can be harmful but they are definitely floating around and prevents CALD parents from taking dietician referrals seriously*…” (FGD 3, Brimbank)

Further, CALD communities viewed their participation in childhood obesity prevention services as something shameful, something to be avoided and as a sign of weakness.

*“…what would people say if they found out their child has obesity*, *and what kind of parents are they since they allow such a thing to happen*, *they have these fears…”* (FGD 1, Greater Dandenong)

City council staff commented that council-initiated obesity prevention efforts were challenged by the denial of childhood obesity found among CALD communities.

“… *There is a lot of blaming in families as to whose fault it is that the child is obese*, *so the easiest option is to deny its existence… it is very hard to design obesity prevention programs when the community is in denial where they will not easily join those programs…*” (FGD 5, Hume)

Dieticians argued that CALD communities often believed that they (dieticians) did not have culturally appropriate knowledge and viewed dietetic services as “unrealistic” and not culturally relevant. Similarly, MCH nurses noted that although CALD communities were familiar with the concept of treatment of disease, they failed to understand the concept of prevention, and viewed the MCH service as a childhood obesity treatment centre and not as an obesity prevention service.

*“…CALD parents are concerned only when they have had a direct experience with childhood obesity with one of their own children…they can’t understand the concept of taking action before something happens…”* (FGD 4, Brimbank)

### Poor utilisation of MCH services by CALD groups

The poor understanding of prevention among CALD groups also led to their poor utilisation of MCH services. MCH nurses indicated that parents are regular in their first few appointments for their first child born in Australia, following which they fail to keep up the schedules, thereby foregoing the opportunities to learn about healthy eating concepts in childhood.

*“…most migrant parents do not come regularly for MCH appointments…it is important to talk about childhood obesity to parents before they get to school*, *as that’s when their children’s eating habits are formed…”* (FGD 8, Maribyrnong)

There was consensus among service providers on CALD parents’ poor MCH service attendance negatively impacting their knowledge of healthy eating behaviours in their children. For instance, playgroup facilitators noted that CALD parents did not know about the introduction of timely solid foods to young children. Similarly, school staff reported that CALD parents were unaware of the concepts of healthy lunch box and portion control. Early years’ service staff stated that despite educating newly-arrived migrant parents on healthy eating, they often insisted on continuing to feed the children according to their cultural food habits.

#### Poor CALD parental role modelling on healthy lifestyle behaviours

Schoolteachers in particular, identified poor CALD parental role modelling on healthy lifestyle behaviours, wherein unhealthy parental behaviours (unhealthy eating and sedentary lifestyle) were followed by their children, despite the ongoing efforts by the school to reinforce healthy behaviours in their students.

*“…because parents are not role-modelling children are bringing unhealthy food to school*, *these are the parents’ practices carrying on through to their kids… we at schools have to work uphill to reinforce concepts of healthy lunch boxes among these children…”* (FGD 3, Brimbank)

Playgroup facilitators indicated that although they educated parents on adopting healthy diets for their children, CALD mums were often resistant to change.

*”…we tell them to stop sending milk bottles with their children attending kindergarten*, *but yet they insist and these children don’t eat solid food but drink milk during the day…”* (FGD 1, Greater Dandenong)

Overall, early years’ service staff and schoolteachers expressed discouragement in their obesity prevention efforts not being supported by parents. However, school nurses disagreed with teachers and early years’ service staff and stated that if parents themselves possessed low levels of knowledge on health behaviours, then they would be incapable of role-modelling and other stakeholders including teachers, doctors and nurses should step in.

”*…we cannot blame these parents if they don’t know about healthy eating*, *and end up as poor role models for their children…ultimately the message needs to be followed… at this stage we need school teachers*, *nurses*, *doctors*, *and early years’ service staff to jump on board and take action…”* (FGD 6, Hume)

#### Intergenerational knowledge gap in healthy lifestyle behaviours among CALD parents

Schoolteachers identified an intergenerational knowledge gap in healthy lifestyle behaviours prevailing among CALD parents. Due to cultural barriers, CALD children were not successful in convincing their parents about the healthy lifestyle knowledge imparted in schools; further due to language and cultural barriers, schools experienced difficulties in engaging parents.

*“…CALD mums are often isolated at home*, *and do not know what is going on in the community or at schools*, *they do not listen to health messages given by their children…also when they try going to the school*, *because of their limited English*, *parents disengage from the school quickly…”* (FGD 7, Maribyrnong)

School nurses emphasised the need to address healthy lifestyle among parents and children together in group sessions, in order to bridge the intergenerational knowledge gap and create an awareness of the importance of healthy lifestyle among CALD parents.

#### Difficulties in health information utilisation among CALD communities

Service providers also identified difficulties in health information utilisation among CALD communities wherein despite the availability of translated materials, community members were not able to understand the healthy lifestyle information provided at MCH and community health clinics and required verbal explanations for changes in their health behaviour.

*“…CALD parents cannot understand the ‘why’ behind fliers or posters*, *why do they need to eat more fruits and vegetables*, *or do exercises everyday…they want someone to explain and convince them what are the reasons for changing their food habits and food choices…”* (FGD 4, Brimbank)

Service providers recognised that the current information provided was not at an appropriate level for the literacy of the community and acknowledged that there is a need for more pictorial resources due to difficulties in understanding textual health information.

*“…most of our clients have low literacy levels*, *so we need more pictorial based resources … now we have lots of textual information but they are not properly used by the clients…”* (FGD 5, Hume)

Since CALD parents often relied on the media and internet for health information, nurses were concerned over the credibility of these sources and their impact on the CALD communities’ health behaviours.

“…*We are only one of the people they (CALD community) listen to*. *There are many others telling them how to be healthy…there are blogs*, *grandma and Dr Google…do they believe information is true if it is written by a hospital?… they can easily get confused*” (FGD 2, Greater Dandenong)

Despite the conflicting sources of health information identified, service providers agreed that in general, they lacked understanding of the CALD communities’ beliefs regarding the sources of health information they valued and their adoption of lifestyle changes based on their beliefs.

*“…we need to find out how they value information … we need to understand whether their health behaviours are based on what they read*, *or what they believe and already know… as of now we don’t know…”* (FGD 7, Maribyrnong)

Overall, service providers acknowledged that the various cultural barriers experienced by CALD communities were not only responsible for their poor engagement in childhood obesity prevention services, but also impeded their (service providers’) obesity preventive service delivery efforts among these communities.

### Service-level barriers in childhood obesity prevention

#### Lack of clear policy guidelines on childhood obesity prevention

Service providers across all services reported on the lack of clear policy guidelines on childhood obesity prevention and the lack of clarity around the various services responsible for it, including staffing and budget constraints. There were differences in service providers’ perception of the primary service responsible for obesity prevention among children; while city council staff identified MCH as the main service involved in childhood obesity prevention, early years’ services staff stated that doctors had the primary responsibility for children’s health and should play a more active role in childhood obesity prevention.

“…*Most doctors wait for an issue to happen and then tackle the problem*, *more focus on prevention is needed… Clinics should have lots of posters for obesity in children and tell us to call a number for free resources on healthy eating*. *…”* (FGD 4, Brimbank)

MCH nurses argued that no weight monitoring was done after the age of four when children entered primary schools. School nurses on the other hand indicated that children’s weight check-up in schools was permitted only when the parent requests for it.

*”… Schools are not expected to check children’s weight routinely…then whose responsibility is it to weigh children? Parents need to first know about child overweight for them to alert the school…”* (FGD 8, Maribyrnong)

Schoolteachers were unsure of the current school policies regarding their role in educating parents on healthy eating.

*“… Should primary school teachers actually provide education on healthy eating to parents? Are we equipped in terms of time and budget…we have no clear policy on this…”* (FGD 6, Hume)

#### Time constraints in MCH services

MCH nurses identified time constraints in MCH services and argued that due to short appointment schedules and the presence of competing priorities, they could not prioritise childhood obesity in their consultations with CALD parents.

*“…we don’t have time during our consultations to talk about childhood obesity as we would like to …”* (FGD 3, Brimbank)

*”… with only 6*.*75 hours they keep adding things*, *health surveillance*, *safe sleeping*, *nutrition messages*, *domestic violence etc*. *… and things are spread thinly*, *so we cannot focus only on childhood obesity*.*”* (FGD 7, Maribyrnong)

#### Inadequate dietetic services

Service providers reported that inadequate dietetic services were a growing problem in their communities with timely consultations for parents being hindered by huge waitlists. While schoolteachers and MCH nurses stated that they give referrals to dietitians in the first instance of identifying overweight in a child, dieticians on the other hand argued that since they faced staffing constraints, parents were often unable to get timely referrals.

“…*especially in the West (Western Victoria) we have huge waiting lists…we don’t have enough time for seeing patients…also most of us are part-time due to lack of adequate funding…”* (FGD 4, Brimbank)

#### Obesity-related stigma

Dieticians and nurses emphasised that obesity-related stigma prevented them from effectively discussing obesity and weight issues in children among migrant and refugee parents, which ultimately affected their delivery of care to these families. Due to their perceived fear of losing contact with these families, MCH and Refugee Health nurses avoided direct discussions with them on the risk of obesity among their children.

*“…we usually do not discuss weight issues with migrant and refugee families…it is a sensitive issue for many of them…”* (FGD 2, Greater Dandenong)

Dieticians added that they refrained from using words such as ‘obesity’ or ‘overweight’ in their consultations with parents, due to their fear of CALD communities’ disengagement from services.

*“…we are reluctant to talk about overweight and obesity as we are afraid that if we offend the family*, *they will disengage from the service*, *as parents have a huge fear if their child is labelled as obese…”* (FGD 4, Brimbank)

#### Lack of childhood obesity data

Service providers also identified the lack of childhood obesity data which undermined their obesity prevention efforts in the community. Health promotion officers and dieticians reported that they were unaware of the current status of childhood obesity in their community as that information was unavailable, and cited the lack of school ‘weigh-ins’ as a key barrier to obtaining childhood obesity data.

*“…children will need to be weighed by schools to have this information and currently there are numerous barriers for doing that*…*”* (FGD 5, Hume)

While school nurses agreed that the lack of annual and mandatory weight checks of children at schools contributed to the lack of childhood obesity data, community health nurses reiterated the need for governments to provide information on childhood obesity rates.

*“…we don’t have current data on childhood obesity levels in each LGA (local government area)…then how can we motivate the community to be more aware when we ourselves do not have this information… this message needs to come from above*, *from the government …”* (FGD 8, Maribyrnong)

Service providers discussed that since CALD communities are not homogenous, the lack of adequate data on childhood obesity led to the failure of identification of high-risk migrant groups, which in turn, they perceived, negatively impacted the development of targeted childhood obesity prevention strategies. Overall, service providers unanimously expressed their discontentment regarding the lack of childhood obesity data provision by the government which hindered their capacity to motivate CALD community members to engage in childhood obesity prevention.

### Proposed changes to current childhood obesity prevention approaches

#### Collaborative approaches between services

Service providers reported on the need for collaborative approaches between services including the removal of silos, better coordination between prevention and treatment, and improved centralised governance on childhood obesity prevention.

”…*we cannot work in silos and not know what the other services are doing… we need a centralised approach with clear roles and responsibilities…”* (FGD 2, Greater Dandenong)

*“…hospitals and clinics should collaborate with community support services to link the prevention with treatment services…”* (FGD 3, Brimbank)

Service providers voiced their concerns regarding the need for an ongoing service for children’s weight monitoring beyond the MCH service key-stage visits which currently ceased when children completed four years.

*“…how to link between MCH and schools? We need some sort of service after MCH to continue weight monitoring of children after the MCH key stage visits…who will that be?”* (FGD 7, Maribyrnong)

Service providers argued that ensuring better coordination between health services and community initiatives would enable their understanding of the existing obesity prevention governance.

#### Community ownership of childhood obesity prevention initiatives

Health promotion officers insisted that enabling the community ownership of childhood obesity prevention initiatives, wherein communities have a role in the co-design and co-development of initiatives was of greater importance as it would empower CALD communities to participate in them. City council staff added that obesity prevention action at a community level as opposed to the systems level would encourage community ownership of initiatives.

“…*Community driven model is welcoming and supportive*, *depending on the service provider model alone is not working… involving community members will improve the community ownership of these programs…”* (FGD 8, Maribyrnong)

*“…there needs to be greater emphasis in building resilient communities …we need to support community members in taking control of their own health…”* (FGD 5, Hume)

Service providers discussed the integration of healthy eating messages within community cooking and gardening classes, and creating opportunities for CALD parents to buy fresh produce from mobile vans stationed at close proximity to schools, during the school pick-up and drop-off times. Service providers emphasised that settlement services had easy access to CALD groups and can be utilised to educate them on adopting healthy lifestyle patterns and accessing the various preventive health programs in the community.

*“…at settlement services*, *migrants can be made aware of the programs running and what they are for…so they feel like participating in them…”* (FGD 4, Brimbank)

*“…having knowledge of the various initiatives during their settlement phase will also help their integration smoothly into the community*…” (FGD 7, Maribyrnong)

Service providers identified that these approaches would not only serve as obesity prevention approaches among CALD groups, but also address the social isolation prevailing among these groups and enable their integration within the host society.

*“…community classes can also be used for community building which is good because they address social isolation…”* (FGD 1, Greater Dandenong)

#### Bicultural workers facilitating CALD engagement in childhood obesity prevention services

Service providers identified bicultural workers as facilitators to the engagement of CALD groups in childhood obesity prevention services. City council staff noted that using bicultural workers as playgroup facilitators can be more advantageous than mainstream staff, especially in encouraging young migrant parents on healthy behaviours. Apart from parental education, early years’ services staff reported on the potential role of bicultural workers to function as links between CALD parents and the MCH service.

*“…we know the importance of well-trained bicultural playgroup staff who can act as healthy lifestyle educators for families… in fact due to their regular contact with parents*, *they can link parents to MCH and other services…”* (FGD 4, Hume)

Service providers recognised that the extremely diverse nature of CALD communities posed difficulties for them in developing information which would be universally suitable across all cultures. However, training members of the CALD community as bicultural workers would enable them to find out more accurately about the information needs of CALD communities.

*“…we need to find out what their understanding of their health is… what would work for them? We need to find the trusted persons in their communities and train them as bicultural workers so they can be key informants of their communities…”* (FGD 3, Brimbank)

Service providers perceived that the CALD community would be able to make the right choices regarding their health when they receive health messages from bicultural workers who were from the same culture and spoke the same language. Contrastingly, schoolteachers reported that employing bicultural workers to engage with CALD parents from all cultures would not be practically possible in a school setting,

*“…we have over 20 different cultures and if you want to train one worker for each culture that is not feasible and we don’t have the capacity to do that in schools…”* (FGD 1, Greater Dandenong)

While service providers in general, supported the valuable role of using bicultural workers in addressing low health literacy among CALD communities, school staff were vocally expressive in acknowledging the complexities involved in creating a bicultural work team, within the existing limited capacity of schools.

## Discussion

This is the first study to explore service providers’ perceptions of key factors responsible for the poor engagement of CALD communities in childhood obesity prevention services, and the key requirements necessary to improve the structure and delivery of childhood obesity prevention services among these communities in Australia. We found that service providers perceived CALD communities to have low levels of health literacy which resulted in their poor engagement with health interventions, a finding shown in other studies as well [49–51]. Health literacy involves cognitive and social skills including cultural and conceptual knowledge, listening, speaking, writing and reading skills which influence the ability of individuals to use information in ways which promote and maintain good health [52,53]. Service providers in our study also perceived CALD communities to have low prose literacy levels due to which they were unable to use textual health information. This finding was supported by two recent Australian studies which indicated that 50.5% of Australians whose first language is not English (vs. 44.3% of Australian-born) fall below level 3 on the prose literacy scale (minimum level for coping with the demands of everyday life and working in an advanced society) [54,55].

We found a mismatch between the health information provided and the information needs of CALD communities. Service providers perceived that CALD communities preferred oral explanations of health concepts as opposed to the mere provision of written health information. Oral literacy or use of the spoken word has been advocated as a key method of transmitting health-related knowledge among CALD groups [56,57]. Service providers in our study emphasised the need for improvement in visual literacy, that is, the use of images to support textual and spoken health information, a strategy which has been successfully used in migrant groups with limited literacy [58]. This finding is not surprising because past studies have shown that the sole dependence on printed health materials without accompanying illustrations, repetition of specific key messages, and affirming the understandability of the provided information with the group, can lead to decreased motivation among ethnic groups from participating in health services [59–62]. Service providers were concerned about the sources of health information sought by CALD parents and the credibility of these sources, as many of them relied heavily on internet sources with misinformation being the chief concern. Studies have shown that CALD groups often rely on the internet, television and radio to obtain health-related information which could be an important contributing factor to their choices regarding lifestyle behaviours [43,63,64]. Further, we found that service providers acknowledged their lack of knowledge on the culturally mediated influences on health literacy which challenged their obesity prevention efforts in the community, this finding was in agreement with other previous studies [44,65].

Low health literacy levels among CALD communities played a pivotal role in unhealthy lifestyle behaviours due to which these communities adopted unhealthy lifestyle patterns. Evidence shows that poor health literacy is associated with poor preventative health behaviours and health outcomes [29,66]. Further, we found that CALD communities’ low health literacy levels also negatively impacted their parental role modelling behaviours, where the unhealthy practices at home interfered with children’s adoption of healthy behaviours taught in schools, a finding shown in other studies as well [67–69]. Additionally, service providers reported on the intergenerational knowledge gap on healthy lifestyle behaviours found among CALD parents which prevented their effective engagement in school-based obesity prevention activities. Renzaho et al [31] have shown that the intergenerational gap in migrant families is due to the differences in the rate of acculturation between parents and children, with children acculturating much faster and adopting the host society’s lifestyle behaviours compared to their parents. Studies have shown that if childhood obesity is only addressed in schools without corresponding parental health education to bridge the intergenerational knowledge gap, successful achievement of program outcomes is unlikely [44,70,71].

Service providers in our study perceived CALD groups to have a poor understanding of the concept of prevention which influenced their motivations to participate in childhood obesity prevention initiatives such as MCH services. Studies have shown that although health practitioners view CALD groups as having poor interest in preventive health care, their limited participation in preventive health interventions has been attributed to their low health literacy levels [72–74]. We also found that poor preventive service participation including MCH clinic attendance by CALD parents led to their lack of knowledge of childhood obesity prevention concepts such as the timely introduction of solid food, healthy lunch boxes and portion control. Additionally, staffing and funding inadequacies in the current dietetic and MCH services, further impeded the utilisation of these services by CALD communities. Service providers in our study lacked the knowledge and skills to address the various cultural barriers experienced by CALD communities in acknowledging childhood obesity as an issue of concern. The existing literature shows that cultural awareness is a prerequisite to achieving cultural competency and implementing childhood obesity prevention initiatives without addressing the cultural barriers will result in culturally incompetent program delivery and poor engagement by CALD communities [8,15,20,75].

This study showed that service providers reported on the cultural misconceptions surrounding the cause of childhood obesity including the belief that obesity is solely influenced by genetics rather than lifestyle factors. Additionally, studies have shown that CALD groups’ participation in obesity prevention services are impacted by their cultural beliefs around the causes of childhood obesity [30,76]. Evidence shows that causal beliefs on illness are known to impact participation in health services and affect treatment outcomes [77–79]. In particular, the mismatch between the beliefs of ethnic groups and Western health practitioners can act as a barrier to the health service utilisation of these groups [77–79]. Incorporating the causal beliefs of CALD groups in the design of culturally-appropriate strategies has the potential to improve their health program participation [80]. Other key factors considered important in the development of culturally appropriate obesity prevention strategies include, improving health literacy levels among CALD parents, addressing the intergenerational acculturation gap, providing adequate linguistic support, maximising community ownership of programs using community health workers and bridging the gap in beliefs and attitudes towards childhood obesity between the health providers and CALD communities [81–83].

Apart from the cultural barriers, we found that compared to mainstream populations, service providers avoided the discussion of childhood obesity with CALD groups due to the presence of obesity-related stigma prevailing among these groups, which undermined their efforts in providing equitable care to CALD groups. Similarly, a systematic review showed that service providers often modified their approach with immigrant patients due to fears of raising sensitive issues related to pre-migratory traumatic experiences and adopted a superficial practice of care, resulting in several underlying barriers to healthy lifestyle adoption among migrant populations, not being addressed [84].

Our study showed that the lack of clear policy guidelines on childhood obesity prevention caused miscommunication between service providers and lack of clarity on the delineation of their roles and responsibilities towards addressing childhood obesity, which led to the formation of silos at both systemic and community levels. Overall, service providers highlighted the need for collaborative approaches between the medical and social models of health, including coordination between prevention and treatment services, and improved community ownership of initiatives. They proposed the incorporation of healthy eating concepts within community-level initiatives and capacity-building of settlement service staff to educate CALD groups on the available obesity prevention services, which would not only foster healthy lifestyle behaviour among these groups but also facilitate their integration into the host society. The existing literature shows similar findings which involve stakeholders from outside the health sector to adopt a multi-level coordinated approach to childhood obesity prevention [85–87]. Service providers in our study identified the lack of continuity in the current MCH childhood obesity monitoring beyond the age of four years, which also affected the availability of childhood obesity data.

Our study showed that across all services there was consensus on the lack of data on childhood obesity. Absence of epidemiological obesity data is reflective of the lack of prioritisation of childhood obesity surveillance by the local and state governments in Australia [88,89]. Contrastingly, population-level childhood obesity surveillance has been prioritised in several developed countries. The WHO European Childhood Obesity Surveillance Initiative in 2006 ensured the collection of school-aged children’s BMI across 13 European countries [90]. Further, governments in USA and UK have mandated routine, regular BMI data collection systems at school levels. The only obesity data currently available in Australia are from population health surveys which report on the nation-wide prevalence of childhood overweight and obesity every three years [2]. However, this data is insufficient to inform the progress of current obesity interventions or their accurate evaluation and demonstration of successful outcomes [91,92].

Despite the establishment of robust school-based obesity surveillance systems in other developed countries, we found that according to the current school policies in Australia, school staff were forced to rely on parental perception of overweight and obesity in order for children to be weighed in schools. With CALD parents often being in denial of overweight and influenced by the stigmatisation of obesity, they were highly unlikely to request school ‘weigh-ins’. Evidence shows that parents from CALD backgrounds were more likely to misclassify their child’s weight [31,93,94]. Schools have been cited as one of the most conducive environments for collecting data on weight and height among young children, and collaborative approaches involving schools, health systems and the government can improve the success rate of school-based childhood obesity monitoring programs [88,89,91].

We found that service providers advocated the use of bicultural workers to evaluate the current levels of healthy lifestyle knowledge among CALD groups and the kind of information these groups would require, in order to tailor health information materials according to their literacy levels. The existing literature shows that bicultural workers provide an ethno-cultural perspective of childhood obesity prevention among CALD groups and enable an understanding of the information needs of these groups [44,81]. Service providers in our study also recommended the training of bicultural workers as playgroup facilitators to enable them to disseminate health messages to CALD groups and function as links between MCH services and CALD parents. Given the rising immigration rates from developing countries into Australia, the bicultural workforce needs special attention regarding their inclusion into the existing service provider workforce. Bicultural workers would also facilitate the tailoring of health programs to accommodate the literacy needs and cultural norms of the CALD groups. Being representatives of the cultural community they originate from, bicultural workers have the potential to influence health behaviour change within their respective ethnic communities through trust-building, and act as a link between the health system and these communities [81,82,95,96].

### Strengths and limitations

We collected data from service providers in four disadvantaged areas and across a diverse range of services which improves the generalisability of study findings to childhood obesity prevention among CALD groups in other disadvantaged areas. The participants were not randomly selected so there may be some selection bias in participant recruitment affecting the responses provided. However, the consistency in themes across the four areas highlight the representativeness of service providers’ perspectives and strengthen the study findings. We were not able to recruit general practitioners and clinicians in this study due to their time constraints in focus group participation, and given their key role in addressing childhood obesity, we consider this as a limitation of this study. There were more female than male participants in this study, however given the predominance of female employees such as nurses, teachers, child care staff and playgroup leaders in the health services and early years’ services, we were unable to achieve gender balance in our study sample. Despite this imbalance, we were not able to detect any differences between the perspectives of male and female participants in our study.

As we have conducted numerous previous studies examining the views of target CALD groups in obesity prevention and health service utilisation [30,31,44,81,97–102], we conducted this study with the sole purpose of obtaining the views of service providers in the delivery of obesity prevention services among CALD groups. This study has uncovered the various barriers impacting the effective delivery of obesity prevention services among CALD groups and the key areas of improvement needed to address these barriers. Nevertheless, we cite the lack of simultaneous collection of data from CALD groups in this study as a limitation.

## Conclusion and Policy Implications

This study has highlighted the key factors influencing the engagement of CALD groups in obesity preventive services, including low CALD health literacy and co-existing deficiencies in the structure and delivery of obesity prevention services. Capacity-building of the bicultural workforce to improve health literacy among CALD groups and facilitate the CALD communities’ ownership of childhood obesity prevention initiatives, is recommended. Adequate cultural competency training of service providers is urgently needed in order to achieve the design and delivery of culturally tailored obesity prevention initiatives. Further, service providers need to be educated on CALD communities’ pre-migratory health service experiences and health conditions, in order to avoid a superficial delivery of care which fails to address the underlying barriers unique to the migrant population. Ongoing childhood obesity surveillance through robust school-based obesity monitoring systems including the training of school staff to maximise data consistency and quality, is crucial to the evaluation of evidence-based interventions. The existence of childhood obesity surveillance data will also enable the identification of high-risk groups and the development of targeted interventions which will ultimately carry the potential of reducing obesity-related disparities.

Obesity prevention needs to be supported by policy changes from all levels of the government; the Federal government to implement changes to the environmental and food industry ‘upstream’ factors; the State government to coordinate the development of integrated approaches tackling behavioural modification ‘midstream’ factors in educational and community services; and finally the local government to design and implement interventions targeting ‘downstream’ or individual-level factors among high risk groups. Due to various cultural, linguistic and religious barriers impacting the social integration of migrants and their effective community participation, culturally congruent multidisciplinary approaches need to be implemented to address these barriers at individual, community and systems levels. With obesity contributing to the second highest burden of disease in Australia, collaborative approaches between health systems, immigrant services, early years’ services and community health services will go a long way in effectively addressing the disproportionate burden of childhood obesity among disadvantaged communities.

## Supporting Information

S1 File Focus Group Schedule. (DOCX)
