# Supplementary material for: Exploring Service Providers' Perspectives in Improving Childhood Obesity Prevention among CALD Communities in Victoria, Australia
Source: PLoS One. 2016 Oct 13;11(10):e0162184. doi: 10.1371/journal.pone.0162184 (PMC5063376; doi:10.1371/journal.pone.0162184)
Supplement: S1 File — (DOCX) [file pone.0162184.s001.docx]

**S1 file: Focus Group Schedule**

1. Do you feel that obesity in children is a health issue that is being adequately addressed in your community? (Probes: role of government/health system/ community/stakeholders)
2. What are your views regarding the current childhood obesity prevention initiatives available to CALD communities? (Probes: current initiatives you are involved in/ or which is in your area of work)
3. What are your views regarding the current utilisation of these childhood obesity prevention initiatives by CALD communities in your area? (Probes: problems they have in accessing our utilising services)
4. What are the various factors influencing the delivery of childhood obesity prevention initiatives to CALD communities? (Probes: various constraints at the service/program; structure/ setting, mode of delivery)
5. What problems do you experience in working with CALD communities for the prevention of childhood obesity? (Probes: language/cultural barriers/ referrals/follow-up appointments)
6. What do you think is missing from the current childhood obesity prevention service in Victoria?
7. What do you think can be done to improve the existing obesity prevention initiatives?
